# Detection of neuronal signatures by means of data-driven tomography

**DOI:** 10.1186/1471-2202-14-S1-P309

**Published:** 2013-07-08

**Authors:** Carlos Aguirre, Eduardo Serrano, Pedro Pascual

**Affiliations:** 1 GNB Department of Computer Science, Universidad Autónoma de Madrid, Madrid, 28049, Spain

## 

Time-frequency tomograms have been used for denoising and component separation of neuronal signals [1]. Time-frequency tomograms are particularly appropriate to identify the time unfolding of the frequency features of the signals. However there are components of neuronal signals, as the neural signatures, that are not well represented by a clear spectral pattern. In this case, a new kind of tomographic transform has been recently proposed, the data-driven tomography[2]. In particular, if in the linear combination of the tomographic operator B (µ, ν) = µt+νO, one chooses an operator O, that is specially tuned to the features of the component that one wants to extract, then, by looking for the particular values of the set (µ = cos(θ), ν = sin(θ)) where the noise effects might cancel out, we may separate the information of very small signals from large noise and also obtain reliable information on the temporal structure of the signal.

**Figure 1:**
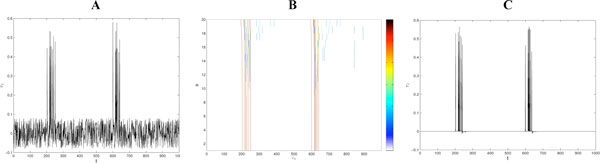
**A. Neuronal signal with neuronal signatures**. **B **Tomogram for 20 values of parameter θ. **C **Signal reconstruction for θ = 8π/40 and projecting from coefficients 230 to 270 and 600 to 640.

We have generated a tuned operator from a typical set of neuronal signatures represented as a firing pattern inside a neuronal burst, we have then applied this data-driven operator to a neuronal signal obtained from a phenomenological model that allows spiking, bursting, sub-threshold oscillations and neuronal signatures, we have also added a uniform noise to the neuronal signal. In Figure 1A the plot of the neural signal with noise is depicted, the neural signature is present inside both bursts.

In Figure 1B the plot color of the tomogram (higher values in red) is built for 20 different values of the parameter θ at intervals π/40. We can see a set of high value coefficients concentrated in the 230 to 270 and 600 to 640 indexes suggesting the presence of the neural signature in both ranges of values. In Figure 1C the two neuronal signatures are extracted from the noisy original signal by projection for θ = 8π/40 from the higher value coefficients.
